# Supplementary material for: Thermal proteome profiling in bacteria: probing protein state in vivo
Source: Mol Syst Biol. 2018 Jul 24;14(7):e8242. doi: 10.15252/msb.20188242 (PMC6056769; doi:10.15252/msb.20188242)
Supplement: Supplementary file 1 — Expanded View Figures PDF [file MSB-14-e8242-s001.pdf]

Expanded View Figures

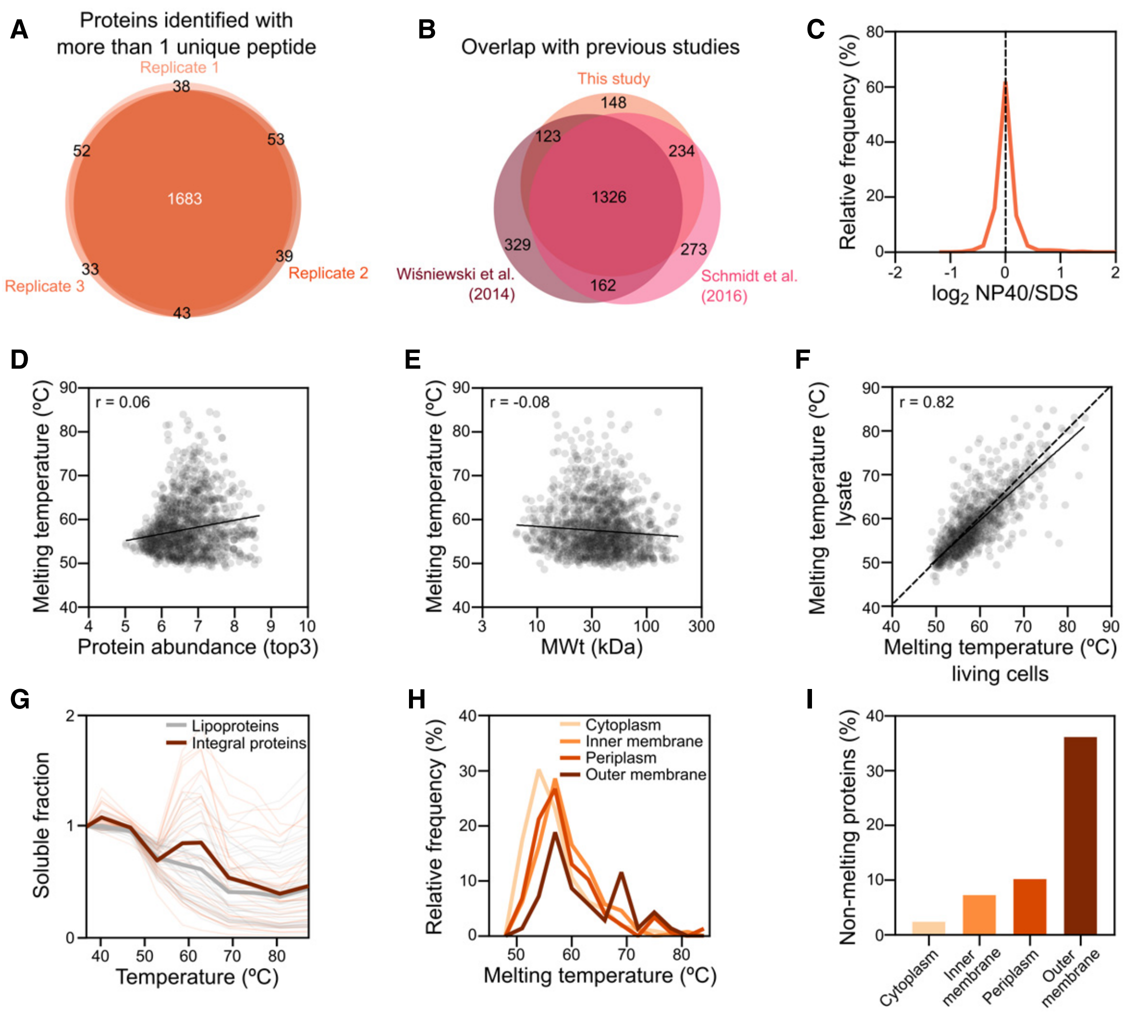

Figure EV1. Melting behavior of proteins identified in the _Escherichia coli_ meltome and their properties.

- A Reproducibility of identified proteins in each replicate of _E. coli_ meltome analysis.
- B Overlap of identified proteins with previously published proteomics datasets obtained from _E. coli_.
- C Distribution of differences between protein abundance after being extracted with NP-40 or with SDS.
- D, E Correlation of melting point with (D) protein abundance (r = 0.06, P = 0.015, as measured by the top3 intensity corresponding to the lowest temperature) and (E) molecular weight (r = −0.08, P = 0.0009).
- F Correlation of melting point in living cells with melting point in lysate—both from TPP (r = 0.82, P < 0.0001).
- G Melting curves for _E. coli_ outer membrane proteins. The average melting curve for each class of outer membrane proteins is shown.
- H Distribution of melting temperatures (T_m) of the _E. coli_ proteome according to their cellular compartment.
- I Fraction of proteins with T_m > 87°C (the highest temperature tested) in each cellular compartment.

© 2018 The Authors

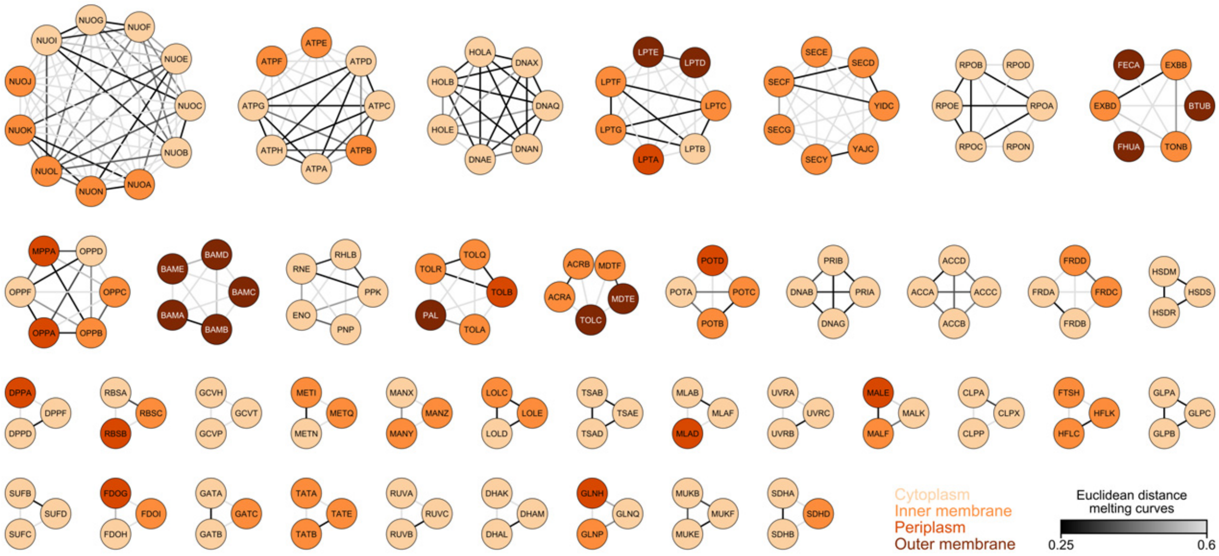

Figure EV2. Melting behavior of protein complexes.

Euclidean distance between all the pairs of melting curves of proteins from each complex—collected from EcoCyc v.21.1 (https://ecocyc.org/; Keseler *et al*, 2017). Only proteins detected with at least two unique peptides in at least two replicates are shown. Node color represents protein location, and edge color represents the average Euclidean distance between the melting curves of complex members.

Figure EV3. Effects of Δ*tolC* on protein thermostability and abundance.

A, B Interaction network of TolC color-coded by the (A) abundance score or (B) stability score. Network was obtained from STRING database by querying only the statistically significant hits (in both abundance and stability) and their interactions with a confidence score of > 0.4.

C Growth of Δ*surA*Δ*tolC* in the absence or presence of 1 mM $MgSO_4$ and 0.1 mM $CaCl_2$ in LB medium containing 4 mM sodium citrate with or without 30 μg/ml kanamycin. Viability was determined by spotting serial dilutions (10^0–10^{-6}) of overnight cultures.

D Cell growth (as measured by OD_{595}) in LB supplemented with 600 mM NaCl of WT, Δ*tolC*, Δ*surA*, and Δ*surA*Δ*tolC* from a starting culture at OD_{595} = 0.2.

E Growth of WT, Δ*tolC*, Δ*surA*, and Δ*surA*Δ*tolC* in increasing concentrations of NaCl in LB. Viability was determined by spotting serial dilutions (10^0–10^{-6}) of overnight cultures.

F Cell growth (as measured by OD_{595}) after 8 h in the presence of aztreonam in WT, Δ*tolC*, Δ*ompF* and Δ*ompF* Δ*tolC* cells (n = 4; error bars represent standard deviation).

G Cell growth (as measured by OD_{595}) after 8 h in the presence of aztreonam in WT, Δ*tolC*, Δ*micF*, and Δ*micF* Δ*tolC* cells (n = 4; error bars represent standard deviation).

H Target engagement affinity of aztreonam in WT and Δ*tolC* cells, measured by thermal proteome profiling compound concentration range (TPP-CCR). Stabilization of the secondary known target of aztreonam (MrcA) is shown.

© 2018 The Authors

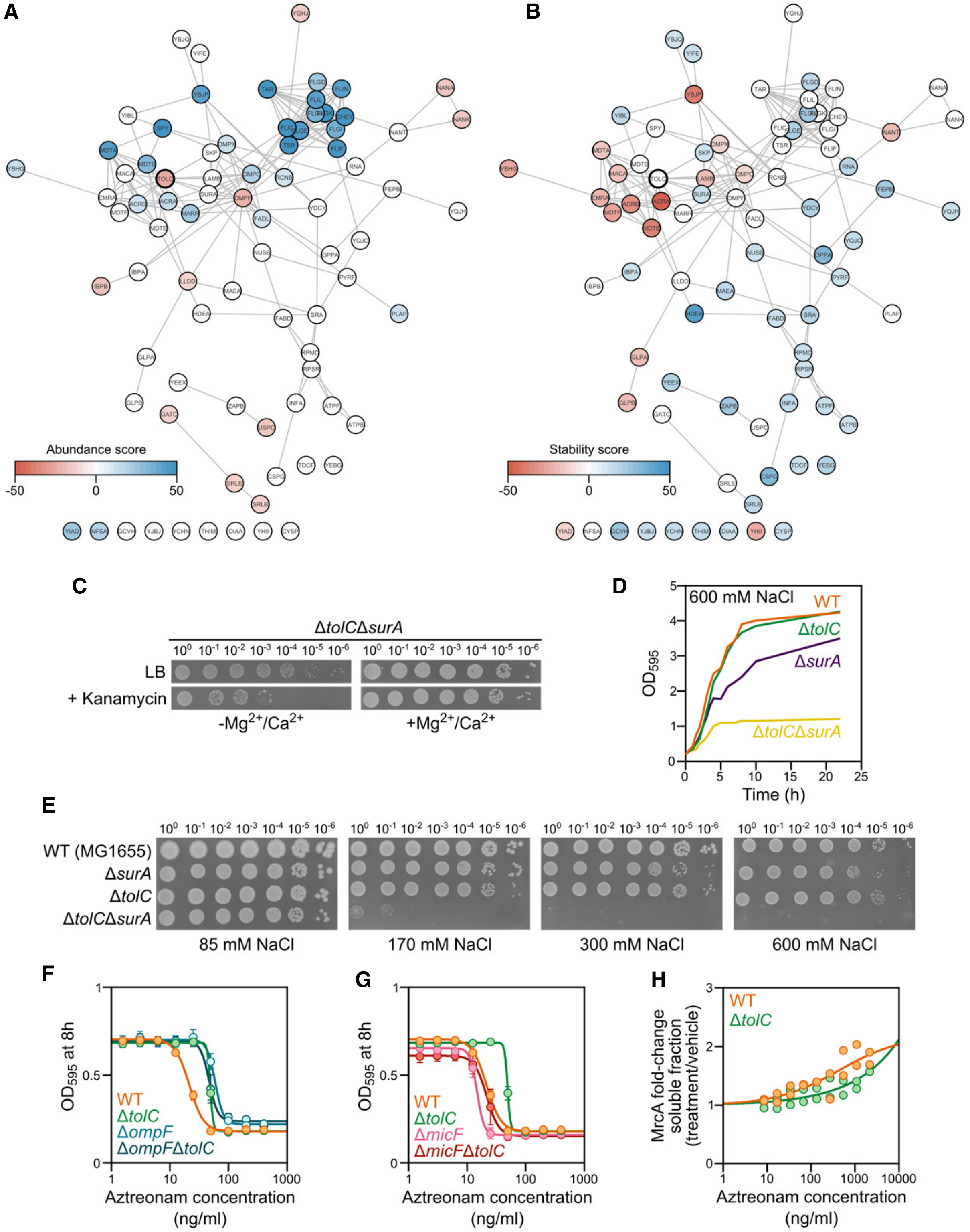

Figure EV3.

© 2018 The Authors

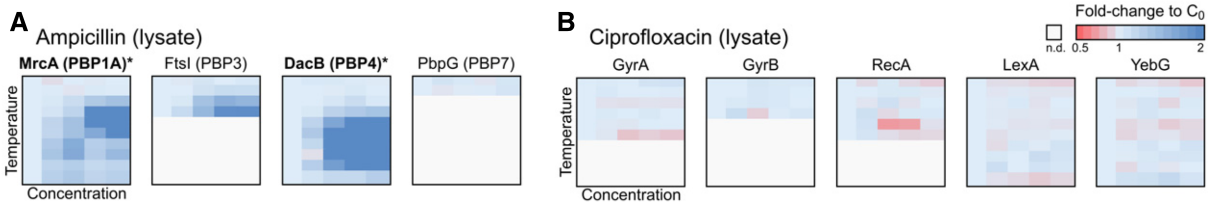

Figure EV4. Identification of direct targets of ampicillin and ciprofloxacin.

A, B Heatmaps of effects on thermostability of proteins after treatment with (A) ampicillin and (B) ciprofloxacin in lysate, with coloring according to what is described in Fig 5A. *FDR controlled at 1% using a bootstrapped permutation approach.

© 2018 The Authors
